# Two novel protein chips for the detection of antibodies against porcine parvovirus

**DOI:** 10.1186/s12917-020-02280-z

**Published:** 2020-02-14

**Authors:** Yue Wu, Xudan Wu, Jinxiu Hou, Xiongnan Chen, Xiaobo Huang, Bin Zhou

**Affiliations:** 1 MOE Joint International Research Laboratory of Animal Health and Food Safety, College of Veterinary Medicine, Nanjing Agricultural University, Nanjing, 210095 China; 2 College of Veterinary Medicine, Yangzhou University, Yangzhou, 225009 China; 3 College of Veterinary Medicine, Sichuan Agricultural University, Chengdu, 611130 China

**Keywords:** Protein chip, Porcine parvovirus (PPV), Antibody detection, Clinical serum

## Abstract

**Background:**

PPV is one of the most important pathogens causing porcine reproductive disorder. It has been shown in clinical cases to be a commonly mixed infection with other important swine diseases which can aggravate the severity of the disease and bring serious economic losses to the pig industry. Serological methods, such as hemagglutination inhibition assays (HAI), serum neutralization (SN), and the modified direct complement-fixation (MDCF) test were utilized earlier, whereas the enzyme-linked immunosorbent assay (ELISA) is the most frequently applied assay to detect PPV-specific antibodies.

**Results:**

We establish the visible protein chip and the cyanine dye 3 (Cy3)-labeled protein chip to detect the clinical serum from pigs. In this study, the recombinant protein VP2 of PPV was expressed in *E.coli*, purified with nickel magnetic beads, and then printed onto epoxy-coated glass slides for preparation of the protein chip. After a series of experiments, the conditions of antigen protein concentration, incubation time of primary antibody or secondary antibody, and optimal serum dilution fold were optimized, resulting in a successful visible protein chip and Cy3-labeled protein chip. The results showed that the positive serum, diluted up to 6000-fold, can be detected by the visible protein chip, and the positive serum, diluted up to 12,800-fold, can be detected by the Cy3-labeled protein chip, suggesting the high sensitivity of these protein chips. Moreover, the positive detection ratio, sensitivity, and specificity of these two kinds of protein chips were higher than those of commercial ELISA antibody detection kits.

**Conclusion:**

Overall, these two protein chips can be used to rapidly diagnose clinical samples with high throughput.

## Background

Porcine parvovirus (PPV) is a non-enveloped, single-stranded linear DNA virus, which belongs to the genus *Parvovirus* within the family *Parvoviridae;* the virus is round or hexagonal in shape [1, 2]. Porcine Circovirus type 2 (PCV2), and Japanese Encephalitis virus (JEV), which can aggravate the severity of the disease and bring serious economic losses to the pig industry [3]. PPV was first detected in China in 1980 [4].PPV is one of the most important pathogens causing porcine reproductive disorder. It has been shown in clinical cases to be a commonly mixed infection with Pseudorabies virus (PRV), classical swine fever virus (CSFV), Porcine Reproductive and Respiratory Syndrome virus (PRRSV). At present, seven genotypes of PPV have been found in the world, which have some differences in genomes and pathogenicity, and isolated and identified in 2017 [5–10]. PPV1 is the most common type in pigs, which has a higher positive detection rate in Chinese pig herds and is harmful to the pig industry. PPV2 was first isolated in Myanmar in 2001, and PPV2 was also found in China since 2006and often causes pathological changes in the lungs [11]. PPV3, also known as pig Hokovirus (PHoV), has been found in Germany, Romania, Guangxi, Hong Kong and many other zones. From 2006 to 2010, it was found that the PPV4 population in China was 2.09%, and the nucleotide sequence homology with the PPV4 genome from the United States was greater than 99%. PPV5 is closely related to PPV4, but does not contain ORF3, indicating that PPV5 is an intermediate of PPV4 [12]. PPV6 was first isolated in Chinain 2014 [8] and similar to PPV4 [13, 14]. Furthermore, PPV7 was isolated in China since 2017. VP2, the main nucleocapsid protein component of PPV, has hemagglutination activity and can induce neutralizing antibodies. Previous studies have shown that the major antigenic site regions of VP2 are amino acids 60–68, 81–88, 266–275, 351–357, and 398–404, where amino acids 378, 383, and 436 are critical sites to determine PPV virulence [15–18].

Serological methods, such as hemagglutination inhibition assays (HAI), serum neutralization (SN), and the modified direct complement-fixation (MDCF) test were utilized earlier, whereas the enzyme-linked immunosorbent assay (ELISA) is the most frequently applied assay to detect PPV-specific antibodies [19, 20]. With the maturity of gene chip technology, the protein chip has also been developed with the features of high throughput, large amount of detection information, rapidity and timeliness, small sample requirements, and high sensitivity [21]. The principle of the protein chip is to regularly immobilize various proteins (such as antigens or antibodies), polypeptide molecules, receptors, and ligands on a certain carrier (such as filters, gels, slides, nano-microspheres, and microplates). On top of this, by adding antibodies labeled with Cy3, Cy5, chemiluminescence, immunogold, etc., the reaction between the antigen and the antibody is amplified to analyze the components that can specifically interact with it [22]. The protein chip is a promising laboratory method in the diagnosis of animal diseases and in the monitoring of disease states, but its application is not extensive. A visible protein chip was established to detect avian influenza (AI), Newcastle disease (ND), infectious bronchitis (IB), and infectious bursal disease (IBD) [23]. The results showed that the sensitivity is significantly higher than traditional detection assays. In Xu et al. [24], a novel protein chip was developed to detect bluetongue virus using a new solid material, iPDMS, as a solid phase carrier, which has a high degree of sensitivity and specificity. Compared with commercial IDEXX BTV ELISA kits, the total, negative, and positive coincidence rates were 95.12, 99.28, and 86.5%, respectively. Additionally, previous publication have described a novel detection platform that leverages horseradish peroxidase (HRP)-mediated silver precipitation within antibody immobilized porosity tuned polyethylene glycol diacrylate (PEGDA) hydrogel microparticles with the operational advantages of suspension arrays for sensitive quantification of biomarkers [25].

Herein we present a visible protein chip detection microarray and Cy3-labeled protein chip detection microarray for the detection of PPV antibodies in pig serum. In this study, we use epoxy-coated glass slides as diagnostic material. Additionally, chemical amplifications using Au nanoclusters or nanoparticles as catalytic centers have primarily been utilized for the silver enhancement/amplification reaction [26]. Cyanine dye 3(Cy3) was also applied to detect antigen–antibody reaction products specifically, which is a procedure that was applied relatively early to the field of protein chips; furthermore, Cy3, Cy5, and IR800 can also be used to label protein chips [27, 28]. Both of these methods were applied to evaluate the seroprevalence of PPV in serum samples collected from pig herds in China.

## Methods

### Virus and cells

PK-15 cells were purchased from ATCC (CCL-33), and propagated in Dulbecco’s Modified Eagle’s Medium (DMEM, GIBCO) supplemented with 10% fetal bovine serum, 100 μg/mL streptomycin, and 100 IU/mL penicillin (GIBCO, Invitrogen). The attenuated strain, NADL-2, of PPV was obtained from the National Institute of Veterinary Drug Control.

### Gene synthesis and plasmid construction

According to the advanced structure and GC content of the PPV VP2 gene sequence (JQ710896.1) and the codon usage frequency of the *Escherichia coli* host, the VP2 optimized gene sequence (amino acids 156–438) was optimized using software of Rare Codon Caltor and synthesized by Wuhan Jinkaili Bioengineering Co., Ltd. Hind III and BamH I restriction sites were introduced at the 5′ and 3′ ends of the VP2 optimized gene (amino acids 156–438), respectively. The pCold vector and the VP2 optimized gene fragment (amino acids 156–438) were double digested and ligated with T4 ligase. The ligation product was transformed into the *E.coli* DH5α competent state and cultured at 37 °C for 12 h. The selected positive clones were cultured in IR800 medium for 12 h. Plasmids were extracted and sequenced. The correct plasmid was identified as pCold-VP2.

### Expression and purification of PPV VP2 protein

Rosetta2 (DE3) cells (Novagen, Shanghai, China) transformed with pCold-VP2 were cultured in LB medium supplemented with 100 U/mL penicillin and 100 μg/mL streptomycin, were grown at 37 °C until achieving the logarithmic phase (at OD600 of 0.6), and induced by Isopropyl-thio-galactoside (IPTG) at a final concentration of 0.1 mM for 16 h at 18 °C. The re-suspended cells were lysed by sonication on ice for 8 × 4 s with 3 s intervals. The lysate was centrifuged at 10,000 g for 15 min at 4 °C, and the supernatant and the pellet resuspended in PBS were both analyzed by SDS–PAGE to observe the solubility of the target protein. The fusion proteins in the inclusion bodies were denatured using 8 μM urea at 4 °C overnight, and the supernatant was retained after centrifugation. His-tagged VP2 fusion proteins were bound to Ni-NTA resin (Qiagen, Hilden, Germany) and purified following the manufacturer’s instructions. Finally, freshly purified proteins were treated with Detoxi-GelTM Endotoxin Removing Gel (Thermo Fisher Scientific, Waltham, USA) to remove endotoxin derived from bacterial culture in the fusion proteins. Aliquots of purified protein were stored at − 80 °C.

### Production of mouse anti-VP2 polyclonal antibody

One group of Balb/c mice (Qinglongshan Animal Breeding Farm, Nanjing, China) were immunized at 100 μg VP2 protein with Freund’s complete adjuvant (1,1, Sigma-Aldrich, USA) to form an emulsion. Another group infected with the same dose of sterile saline was set as negative control. After the first immunization, the second and third immunizations were performed after Freund’s incomplete adjuvant (1,1) emulsification every two weeks. After the third immunization, serum was collected from the orbital blood and stored in a refrigerator at − 80 °C, similarly as measured in previous study [29]. The mice were sacrificed by cervical dislocation without anesthesia. After confirming the death, the mice were put into a freezer for centralized processing.

### Indirect immunofluorescence (IFA)

PK-15 cells were infected with PPV for 48 h and fixed with 4% paraformaldehyde, permeabilized with 0.1% X-Triton, and blocked with 10% sheep serum. PK-15 cells were incubated with mouse anti-VP2 polyclonal antibody (1500) for 2 h at 37 °C, washed with PBST 3 times, incubated with goat anti-mouse antibody (Alexa Fluor&488, abcam, USA) for 1 h at 37 °C, and washed with PBST 3 times. The nucleus was dyed with DAPI for 20 min and observed with a confocal microscope.

### Antibody labeled

The standard PPV positive antibody (National Institute of Veterinary Drug Control) was conjugated to Cyanine3 fluorescent dye using a Lightning-Link® HRP conjugation kit (Innova Biosciences, UK).

### Protein chip preparation

Before printing, 50% of the moisture of the spotter was prepared at 4 °C. VP2 protein of 0–0.4 mg/mL was printed on epoxy-coated glass slides with 2 × printing buffer (Capital Biochip Biotechnology Co. Ltd., Beijing, China); each dilution of VP2 protein was printed 3 times using the SmartArrayer™ microarray spotter (Capital Biochip Biotechnology Co. Ltd., Beijing, China). The spotted chip was placed in a 37 °C molecular hybridization instrument and hydrated for 10 h. After 5 min washing with PBST, 1% BSA was added to each sub-array for blocking. Then the prepared slides could be used immediately or could be stored at 4 °C after centrifuging and drying. The diluted standard PPV positive antibody serum was added to the chips and incubated for 1 h at 37 °C. After washing and drying, the protein chip was observed by scanning with a Luxscan-10 K/A chip scanner (Capital Biochip Biotechnology Co. Ltd., Beijing, China).

### Establishment of visible protein chip

To determine the optimal concentration of diagnostic antigen, PPV VP2 protein were diluted to 0.002 mg/mL, 0.02 mg/mL, 0.05 mg/mL, 0.1 mg/mL, 0.2 mg/mL, 0.3 mg/mL, and 0.4 mg/mL with 2 × printing buffer, each concentration was diluted 3 times. The blocked chip was added with 100-fold diluted positive control serum and incubated at 37 °C for 1 h. After washing with PBST three times, 100-fold diluted rabbit anti-pig IgG was added on the chips and incubated at 37 °C for 1 h. After washing with PBST three times, the well-mixed silver staining solution A and B (Sigma-Aldrich, St. Louis, USA) were added into each well, and then the results were observed directly. Once the antigen concentration was determined, the optimized concentration of VP2 protein was printed on epoxy-coated glass slides for subsequent experiments. The positive control serum was diluted to 10-, 100-, 1000-, 2000-, 4000-, 5000-, and 6000-fold to determine the optimal serum dilution. The incubation time of primary and secondary antibodies was set to 30 min, 45 min, and 1 h, respectively, and a total of 9 groups of time gradient tests were performed to determine the optimal antigen–antibody reaction time.

### Establishment of Cy3-labeled protein chip

To determine the optimal antigen concentration, PPV VP2 proteins were diluted with a series of concentrations (0.002, 0.02, 0.05, 0.1, and 0.2 mg/mL) with 2 × printing buffer, each concentration was diluted 3 times. The blocked chip was added with 100-fold diluted positive porcine serum and incubated at 37 °C for 1 h. After washing with PBST, 100-fold diluted Cy3-labelled rabbit anti-porcine IgG was added and incubated at 37 °C for 1 h. After washing with PBST for 5 min, the chip was centrifuged and dried. The results were observed by scanning with a Luxscan-10 K/A chip scanner. Once the antigen concentration was determined, the optimized concentration of VP2 protein was printed on epoxy-coated glass slides for subsequent experiments. PPV antibody positive serum was diluted 10–25,600-fold to determine the optimal serum dilution ratio. The incubation time of primary and secondary antibodies was set to 30 min, 45 min, and 1 h, respectively, and a total of 9 groups of time gradient tests were performed to determine the optimal antigen–antibody reaction time.

### Determining the positive–negative threshold and specificity

Thirty known negative sera against PPV were added to the prepared protein chips to determine the cut-off value for antibody detection. In addition, the protein chip was divided into four microarrays, and three spots were repeated in each array. CSFV, PPV, JEV, and PRRSV positive serum were added to the corresponding arrays to verify the specificity.

### Detection of clinic samples

The established PPV visible protein chip was used to detect 9 negative and 42 positive swine serum samples that had been detected using an indirect ELISA assay (Wuhan Keqian Biology Co., Ltd., China). The serum dilution ratio of the ELISA is 2-fold. The established PPV Cy3-labeled protein chip detection microarray was used to detect 15 negative and 19 positive swine serum samples that had been detected by indirect ELISA. Then, 120 clinical swine serum samples (farmer permission) from the pig farms in Jiangsu province were tested using the two established protein chip technologies and compared with commercial PPV ELISA antibody detection kits.

## Results

### PPV VP2 protein preparation and its specificity

**Fig. 1:**
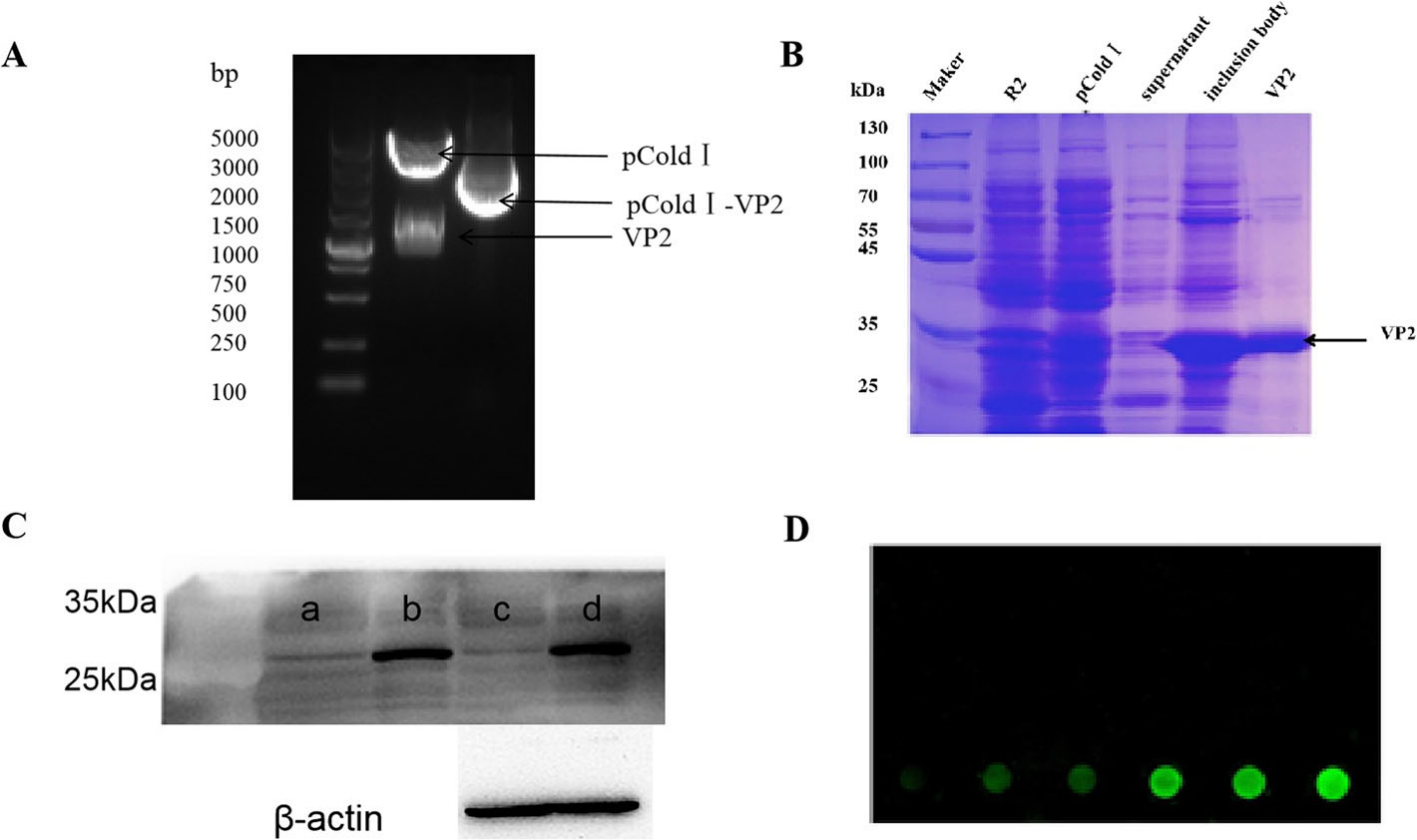
Identification of PPV VP2 protein. **a** Identification of prokaryotic expression vector digested by restriction enzyme identification. **b** Identification of PPV VP2 protein expression and purification by SDS–PAGE. **c** Detection of reactivity of anti-VP2 polyclonal antibodies by Western blotting. a and b. The cell lysate of empty vector and induced pColdI-PPV VP2 plasmid. c and d. The cell lysate of empty vector and transfected pcDNA3.0-PPV VP2 plasmid (**d**) The specificity of PPV VP2 protein verified using the protein chip

The PPV VP2 gene was cloned into the pCold I vector (Fig. 1a). Upon induction by IPTG, PPV VP2 protein was expressed in the inclusion body (Fig. 1b). After the expressed PPV VP2 protein was purified, a mouse anti-VP2 polyclonal antibody with good specificity was successfully prepared. The purified VP2 protein was proved to have good specificity (Fig. 1c) and was used to prepare protein chips (Fig. 1d).

### Protein chip preparation

**Fig. 2:**
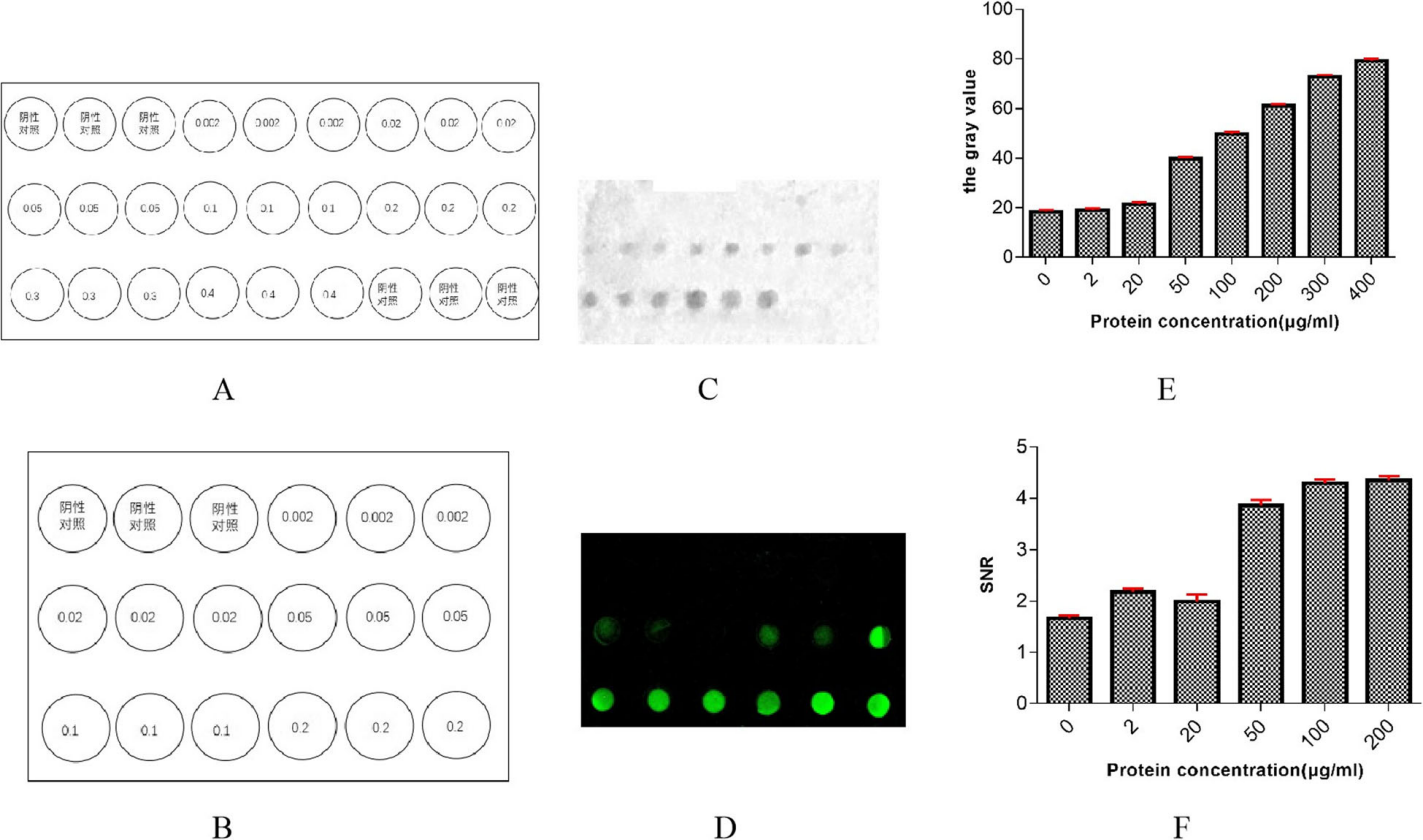
Determination of the optimal spot concentration. **a** and **b** Dot sequence order of both protein chips. **c** and **d** Optimization of the antigen of both protein chips. **e** and **f** The antigen diagnostic concentration of the visible protein chip microarray is optimized. The amount of 0.3 mg/ml can be used as a diagnostic concentration after gray value analysis. The antigen diagnostic concentration of the Cy3-labeled protein chip diagnostic microarray is optimized. The amount of 0.1 mg/ml can be used as a diagnostic concentration

PPV VP2 protein was diluted to 0–0.4 mg/mL with 2 × printing buffer to determine the optimal protein concentration. Preparation of protein chips based on the dot sequence are shown in Fig. 2a and b, respectively; the results showed 0.3 mg/mL as the optimal protein concentration of the visible protein chip detection microarray (Fig. 2c, e), and 0.1 mg/mL as the optimal protein concentration of the Cy3-labeled protein chip detection microarray (Fig. 2d, f).

### Optimization of the serum dilution

**Fig. 3:**
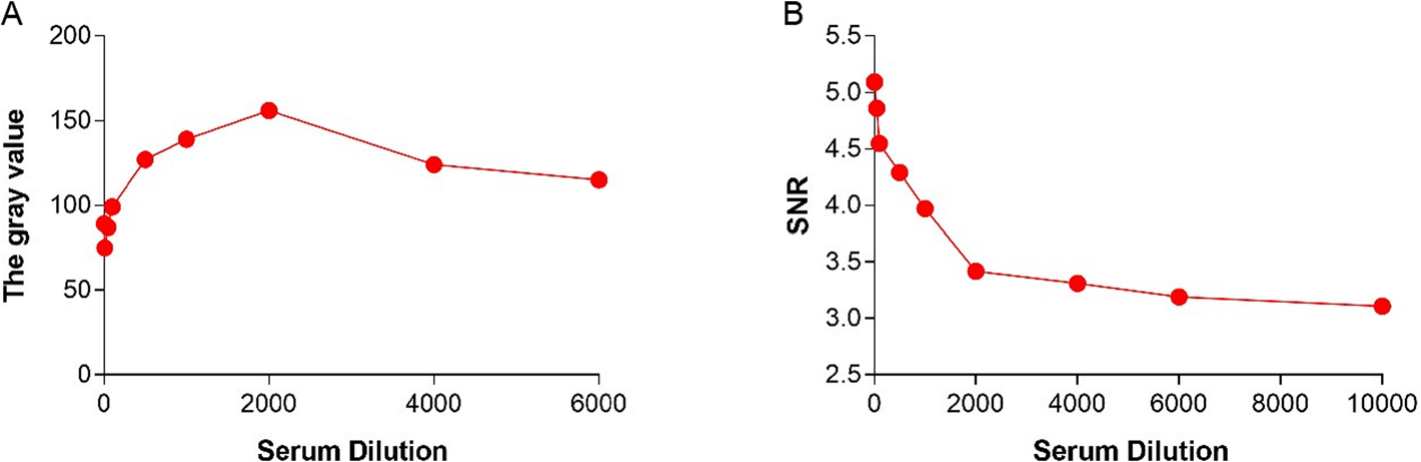
Optimization of the serum dilution. A 2000-fold dilution of serum was determined as a diagnostic concentration of the visible protein chip detection microarray (**a**) and the Cy3-labeled protein chip detection microarray (**b**)

PPV antibody positive serum was diluted 10–25,600-fold to determine the optimal serum dilution ratio. The visible protein chip detection microarray could dilute serum up to 6000-fold, and a 2000-fold dilution of serum was determined as a diagnostic concentration (Fig. 3a). The Cy3-labeled protein chip detection microarray could dilute serum up to 25,600-fold, and it was determined that a 2000-fold dilution of serum was a diagnostic concentration (Fig. 3b).

### Optimization of the incubation time of primary and secondary antibodies

**Fig. 4:**
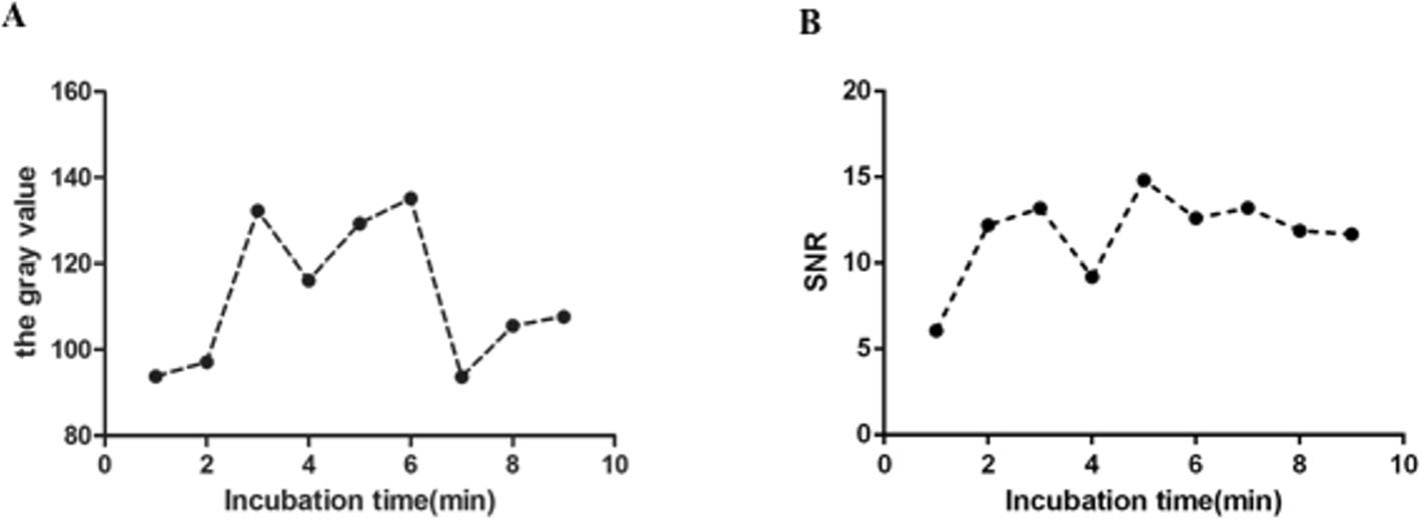
Optimization of the incubation time of primary and secondary antibodies. **a** The optimal incubation time for the visible protein chip detection microarray was 30–45 min for the primary antibody, and 60 min for the secondary antibody. **b** The optimal incubation time for the Cy3-labeled protein chip detection microarray was 30–45 min for the primary antibody, and 45–60 min for the secondary antibody

In order to determine the optimal antibody reaction time, the first and second antibody incubation gradients were set for 30 min, 45 min, and 1 h, respectively, and a total of 9 sets of time gradient tests were conducted at 37 °C in the hybridization apparatus. Other reaction conditions were unchanged. Silver staining or protein chip scanner observations were done. The results showed that the optimal incubation time for the visible protein chip detection microarray was 30–45 min for the primary antibody and 60 min for the secondary antibody (Fig. 4a); the optimal incubation time for the Cy3-labeled protein chip detection microarray was 30–45 min for the primary antibody and 45–60 min for the secondary antibody (Fig. 4b).

### Determining the positive–negative threshold and specificity

**Fig. 5:**
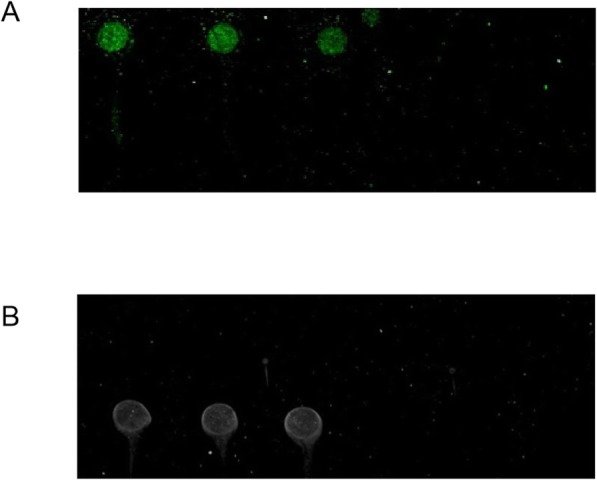
Specificity of protein chip. The CSFV, PPV, JEV, and PRRSV- positive sera were incubated to detect the specificity of the Cy3 protein chip (**a**) or the visible protein chip (**b**). PPV, CSFV, JEV, and PRRSV-positive sera were added to the corresponding microarrays on the chip. As a result, only the microarrays with PPV sera showed the positive signals

When the visible protein chip appeared as dots, the sample could be judged as positive; otherwise, the sample was negative. When the SNR (the ratio of the median value of the signal intensity to the background median value) of the Cy3 fluorescent labeling protein chip was greater than or equal to 3.0, the sample could be judged as positive; otherwise, the sample was negative. The established visible and Cy3 fluorescent labeled chip methods were used to detect clinical CSFV, PPV, PRRSV, JEV positive serum samples identified, The results showed that the visible and the Cy3 labeled protein chip only reacted with PPV positive serum, while CSFV, JEV, and PRRSV serum all showed negative reaction, which indicating that the protein chip detection method established had a high specificity (Fig. 5).

### Coincidence rate of the two protein chips

The established visible protein chip detection microarray was used to detect 9 negative pig serum and 42 positive pig serum that had been detected using commercial PPV ELISA antibody test kits. The results showed that 51 samples were detected using a visible protein chip: 44 were positive and 7 were negative. One of the positive samples detected by the commercial PPV ELISA antibody test kits was detected as negative by the visible protein chip, and 3 negative samples detected by the commercial PPV ELISA antibody detection kits were detected as positive by the protein chip. The negative coincidence rate, the positive coincidence rate, and the total coincidence rate were 85.7, 93.2, and 92.2%, respectively.

The established Cy3-labeled protein chip detection microarray was used to detect 5 negative porcine sera and 19 positive porcine sera that were detected by the commercial PPV ELISA antibody test kits. The results showed that of the 34 detected serum, there were 22 positive samples and 12 negative samples detected by the Cy3-labeled protein chip detection microarray (Table 1). One of the positive samples detected by the commercial PPV ELISA antibody detection kits was detected as negative by the Cy3-labeled protein chip diagnostic microarray, and 4 negative samples detected by the commercial PPV ELISA antibody test kits were detected as positive by the Cy3-labeled protein chip. The negative coincidence rate, the positive coincidence rate, and the total coincidence rate was 91.7, 81.8, and 85.3%, respectively (Table 1). Both the ELISA and the newly established two protein chip detection methods had a certain degree of deviation, resulting in the detection results of the two methods could not reach full compliance. Therefore, we suggested that the test sample can be judged as suspected positive when the visible protein chip dots were not clear and the SNR value of the Cy3 labeled protein chip was between 2.9 and 3.0,

**Table 1:** Coincidence rate of the two protein chips

	Visible protein chip	ELISA	Cy3-labeled protein chip	ELISA
Sample number	51	51	34	34
Positive	44	42	22	19
Negative	7	9	12	15
Positive rate	86.3%	82.4%	64.7%	55.9%
Negative rate	13.7%	17.6%	35.3%	44.1%

### Application of the two protein chips

One hundred and twenty clinical pig serum samples were tested using the two established protein chip technologies and compared with commercial PPV ELISA antibody detection kits. The results show that the positive rate of the two established protein chip technologies was higher than ELISA, and the positive rate of the Cy3-labeled protein chip was higher than the visible protein chip (Table 2).

**Table 2:** Application of the two protein chips

	Visible protein chip	Cy3-labeled protein chip	ELISA
Positive	87	90	85
Negative	33	30	35
Positive rate	72.50%	75.00%	70.80%
Negative rate	27.50%	25.00%	29.20%

## Discussion

Protein chips are widely used for high-throughput proteomic analysis, but the low sensitivity and narrow dynamic range have limited their capabilities in diagnostics and proteomics [21, 30]. Nitrocellulose (NC), polystyrene, poly methyl methacrylate (PMMA), nylon, poly vinylidene fluoride (PVDF), poly-l-lysine (PLL), Poly-electrolyte thin films, and polymer-coated initiator-integrated PDMS (iPDMS) can be used as diagnosis material. Choosing the right substrate for diagnostics is important; here, we use epoxy-coated glass slides as diagnostic material, which have the advantage of being able to efficiently bind to proteins. ELISA requires 200–1000 ng of antigen protein per sample, while the protein chip technology established in this experiment requires only 0.5–2 ng of antigen protein per sample; antigen and antibody can be efficiently bound, so when testing, they only need a micro-scale of serum to be detected. In this study, we set different concentrations of VP2 protein, and we determined 0.3 mg/mL as the optimal protein concentration of the visible protein chip detection micro-array and 0.1 mg/mL as the optimal protein concentration of the Cy3-labeled protein chip detection micro-array. In addition, one PPV antibody-positive pig serum sample was detected as positive when diluted 6000-fold using a visible protein chip, and by 12,800-fold dilution when using a Cy3-labeled protein chip. We ultimately set a 2000-fold dilution as the optimal dilution for the test sera.

As a reasonable incubation time significantly enables efficient binding of antigens and antibodies, the first and second antibody incubation gradients were set for 30 min, 45 min, and 1 h, respectively, to optimize the optimal primary and secondary antibody incubation times. The two kinds of protein chips established in this study are solid-phase protein chips, whereas previous studies also established a suspension protein array for the detection of animal virus antibodies [31]. The two protein chip technologies established have good sensitivity and specificity. In this study, the visible protein chip negative coincidence rate, positive coincidence rate, and total coincidence rate were 85.7, 93.2, and 92.2%, respectively. For the Cy3-labeled protein chip, the negative coincidence rate, the positive coincidence rate, and the total coincidence rate were 91.7, 81.8, and 85.3%, respectively. The specificity and sensitivity of the Cy3-labeled protein chip and the visible protein chip were higher than those of ELISA by about 4.2 and 1.7% respectively, while the Cy3-labeled protein chip was higher than that of the visualized protein chip by about 2.5%. Previous studies about hemagglutination inhibition test (HI) used for the PPV infection showed that the interpretation of HI titers was very complicated and herd size had also a strong effect on HI titers. In contrast to protein chip, HI test still had considerable limited elements [32, 33].

PPV is one of the major pathogens causing sow reproductive failure. It mainly causes reproductive difficulties in recently farrowed sows and skin lesions in newly born piglets. PPV is distributed all over the world and bring a lot of economic losses to the pig industry. At present, there are no effective antiviral drugs against PPV, and the effective control of PPV is still based on immunoprevention. In recent years, protein chips have been increasingly used in fields such as food inspection, proteomics, disease diagnosis, drug screening, agriculture, forestry, animal husbandry, and forensic identification. As a novel laboratory diagnostic technique, the two protein chips established in this study can be used to monitor the level of PPV antibodies in pigs with more sensitive and high-throughput and has important application prospects.

## Conclusion

These two protein chips are used to detect PPV antibodies, and they are highly compatible with commercial ELISA kits in terms of specificity and sensitivity. At the same time, these two protein chip methods also have the advantage of high throughput and have broad prospects in clinical use.

## Data Availability

Raw data is available from the corresponding author on reasonable request.

## Abbreviations

BSA: Bovine Serum Albumin
DAPI: 4′,6-diamidino-2-phenylindole
iPDMS: Polydimethylsiloxane
LB: Luria Broth
PBS: Phosphate buffer saline
